# Development and validation of nomogram models to discriminate between acute aortic syndromes and non-S*T-*elevation myocardial infarction during troponin-blind period

**DOI:** 10.3389/fcvm.2023.1077712

**Published:** 2023-01-20

**Authors:** Fei Tong, Yue Wang, Zhijun Sun

**Affiliations:** ^1^Department of Cardiology, Shengjing Hospital of China Medical University, Shenyang, China; ^2^Department of Obstetrics and Gynaecology, Shengjing Hospital of China Medical University, Shenyang, China

**Keywords:** acute aortic syndromes, non-S*T-*elevation myocardial infarction, D-dimer, cardiac troponin, nomogram

## Abstract

**Background:**

Blood-tes*t-*based methods of distinguishing between acute aortic syndromes (AASs) and non-S*T-*elevation myocardial infarction (NSTEMI) during the troponin-blind period of <2–3 h of symptom onset have not been studied previously. We aimed to explore whether routine biomarkers might facilitate differential diagnosis.

**Methods:**

Data were retrospectively collected from 178 patients with AASs and 460 patients with NSTEMI within 3 h of onset. Differential risk factors related to AASs were identified by univariate and multivariate logistic regression analyses for patients with onset <2 h and onset ≥2 h, respectively, in the cardiac troponin (cTn) cohort. Nomograms were established in the cTn cohort as a training set and validated in the high-sensitivity cTn cohort. To assess the utility of the models in clinical practice, decision curve analyses were performed.

**Results:**

D-dimer, fibrinogen, and age were identified as differential risk factors for AASs with the onset of <2 h. D-dimer at an optimal cutoff level of 281 ng/mL for AASs had a sensitivity of 86.4% and a specificity of 91.3%. A nomogram was developed and validated with areas under the curve (AUC) of 0.934 (95% CI: 0.880–0.988) and 0.952 (95% CI: 0.874–1.000), respectively. D-dimer, neutrophil, bilirubin, and platelet were the differential risk factors for AASs with the onset of ≥2 h. D-dimer at an optimal cutoff level of 385 ng/mL has a sensitivity of 91.8% and a specificity of 91.3%. The AUC of the second nomogram in the training set and the validation set were 0.965 (95% CI: 0.942–0.988) and 0.974 (95% CI: 0.944–1.000), respectively.

**Conclusion:**

Time-dependent quality of D-dimer should be considered for discriminating AASs from NSTEMI. Both nomogram models may have a clinical utility for evaluating the probability of AASs.

## Introduction

Acute aortic syndromes (AASs) are a spectrum of fatal cardiovascular diseases with chest or abdomen pain as the main manifestation, comprising acute aortic dissection (AAD), intramural aortic hematoma (IMH), and penetrating aortic ulcer (PAU) (1). AAD contributes to a high mortality rate of 1–2% per hour in the absence of treatment (2). Given the rare incidence of AASs (1–3) and the non-specific symptoms at presentation, prompt and accurate diagnosis of AASs can be challenging. Non-S*T-*elevation myocardial infarction (NSTEMI) is also a disease with sudden onset chest pain, similar to that of AASs, but with a completely different pathogenesis and therapeutic strategy compared with AASs (3, 4). Guidelines recommend a 0/1 or 0/2-h algorithm to rule out or rule in NSTEMI using high-sensitivity cardiac troponin (HS-cTn) assay if the level of HS-cTn at 0 h is low (4), and thus, a period of 1–2 or 2–3 h from symptom onset are warranted to diagnose NSTEMI with early onset; meanwhile, the troponin-blind interval for the detection of NSTEMI will be further prolonged if the conventional cTn assay is employed (4). There is a pressing need for reliable discrimination between AASs and NSTEMI during the troponin-blind period. The present study aimed to find potential differential risk factors to discriminate AASs from NSTEMI and to develop clinical probability assessment tools amenable to distinguish between AASs and NSTEMI within 1–2 and 2–3 h of chest pain onset, respectively.

## Methods

### Study patients and protocol

This single-center, retrospective study consecutively enrolled 179 patients with AAS and 467 patients with NSTEMI who were admitted to the emergency department (ED) of Shengjing Hospital between January 2017 and June 2022. The inclusion criteria were (1) patients with chest pain diagnosed as AAS or NSTEMI and (2) admission within 3 h of onset. Among them, five patients because of severe infection, two patients because of malignancy, and one patient during pregnancy were excluded from the analysis. This study finally included 178 patients with AAS (149 patients with AAD, 28 patients with IMH, and 1 patient with PAU) and 460 patients with NSTEMI. Of the 638 eligible patients with AAS or NSTEMI, 417 patients from January 2017 to September 2020 were measured by conventional cTnI assays and defined as cTn cohort, 221 patients from October 2020 to June 2021 were measured by HS-cTnI assays and defined as HS-cTn cohort. Diagnosis of AAS was confirmed by computed tomography angiography (CTA). Diagnosis of NSTEMI was confirmed by cTnI above the 99th percentile upper limit and identifiable culprit lesion through early coronary angiography. Blood samples were drawn on emergency admission. cTnI was measured by Beckman Coulter assay; HS-cTnI was measured by Access HS-TnI assay (Beckman Coulter); and D-dimer was measured by HemosIL assay. cTn ratio = absolute value of cTnI/upper reference limit. The study was approved by the Shengjing Hospital of China Medical University Ethics Review Board, and written informed consent was obtained from all patients.

### Statistical analysis

**Figure 1:**
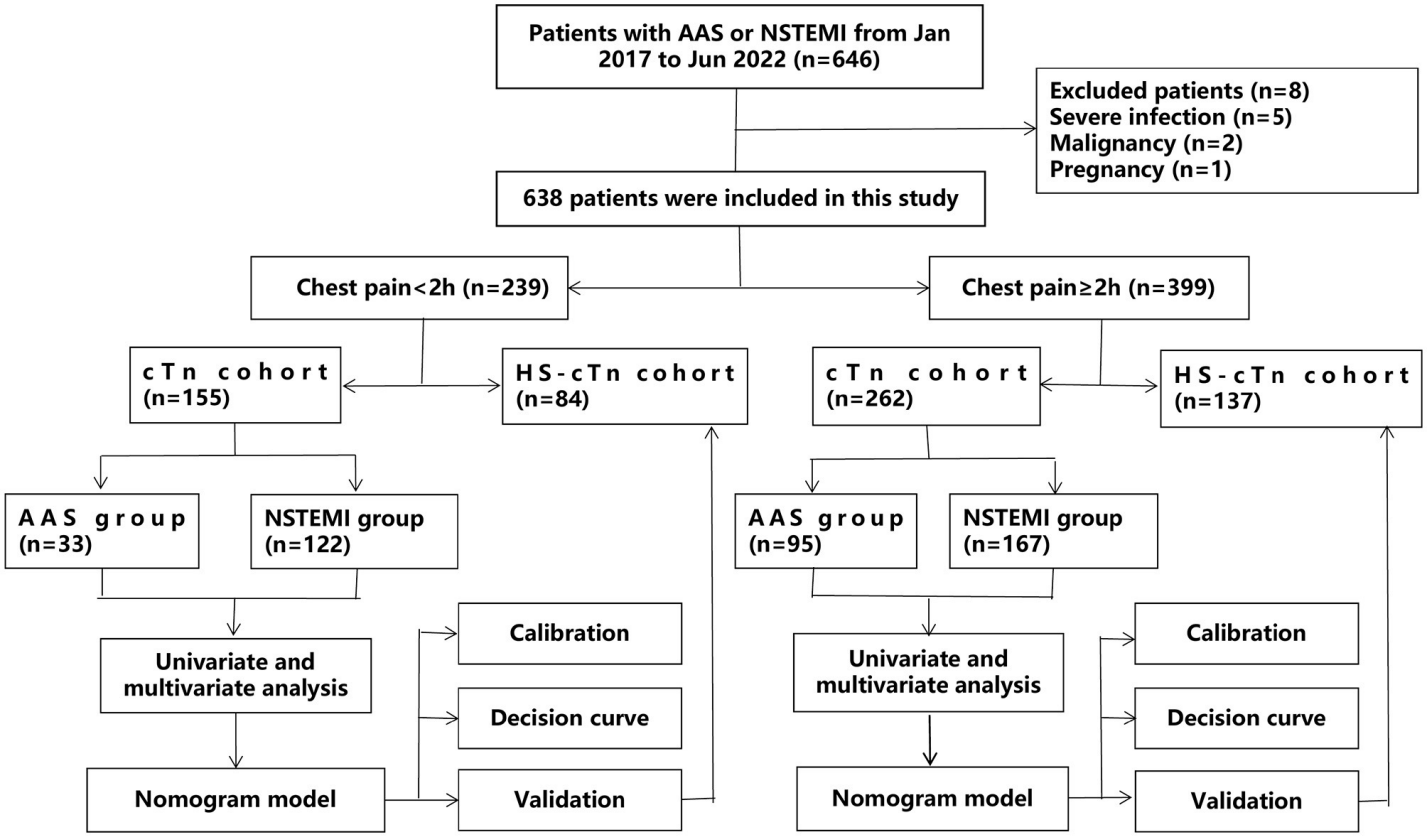
A flow diagram of the overall study design. AAS, acute aortic syndrome; HS-cTn, high-sensitivity cardiac troponin; NSTEMI, non-S*T-*elevation myocardial infarction.

The overall study design is shown in Figure 1. Patients were divided into a subgroup of onset at <2 h and a subgroup of onset at ≥2 h. In the cTn cohort, baseline characteristics between AAS and NSTEMI were compared using the Student *t-*test [if normally distributed continuous variables are presented as mean±standard deviation (SD)], the Mann–Whitney U test (if non-normally distributed continuous variables are presented as median and interquartile range), or the Chi-square test (if categorical variables are presented as frequencies with percentages) in patients with onset at <2 h and patients with onset at ≥2 h, respectively. To identify differential risk factors related to the AAS, we subjected significantly different baseline characteristics for which the univariate logistic regression analysis yielded a *P*-value of <0.05 to a forward stepwise multivariate logistic regression analysis. As the prevalence of AAS in patients with a suspicion of AAS was poorly understood, to ease the generalization of our estimations, we used 25% (i.e., 1 in 4 patients) for calculating the positive predictive value (PPV) and negative predictive value (NPV), as applied in other studies for D-dimer (5, 6).

Nomogram models based on independent predictors were established in the cTn cohort as a training set. Validation of nomogram models was evaluated by bootstrap analysis using 1,000 replications and performed in the independent HS-cTn cohort as a validation set. To assess the calibration of the models, fitted logistic calibration curves were depicted. The areas under the curve (AUC) were calculated to evaluate the discrimination of the models. Moreover, to assess the utility of the models in clinical practice, decision curve analyses were performed. The decision curves were used to quantify the clinical utility of the models, displaying standardized net benefit against risk threshold probability.

Statistical analysis was performed using SPSS (IBM SPSS Statistics 24.0) and R language (version 4.0.3). All hypothesis tests were two-sided, with a significance level of *P* of < 0.05.

## Results

### Demographic characteristics and analysis for AAS patients and NSTEMI patients with chest pain onset of <2 h

Of all the 638 patients, 44 patients with AAS and 195 patients with NSTEMI were enrolled with an onset time at <2 h. In the cTn cohort, this subgroup consisted of 33 patients with AAS (25 patients with AAD, 8 patients with IMH) and 122 patients with NSTEMI. The subgroup in the HS-cTn cohort consisted of 11 patients with AAS (8 patients with AAD, 3 patients with IMH) and 73 patients with NSTEMI. The specific characteristics of patients in the cTn cohort and HS-cTn cohort are reported in Table 1. Of the differential risk factors for AAS by univariate analysis in the cTn cohort, D-dimer, fibrinogen, and age remained significant in the multivariate analysis that included BIL, platelet, and cTn ratio (Table 2).

**Table 1:** Baseline characteristics of AAS *vs*. NSTEMI with chest pain onset <2 h.

**Characteristic**	**HS-cTn cohort** (***N** =* **84)**		**cTn cohort** (***N** =* **155)**		
	**AAS (*****N** =* **11)**	**NSTEMI(*****N** =* **73)**	**AAS (*****N** =* **33)**	**NSTEMI(*****N** =* **122)**	***P*** **-value**
**Demographic data**					
Male sex	11 (100.0%)	55 (75.3%)	26 (78.8%)	92 (75.4%)	0.686
Age (years)	49.5 ± 10.5	62.0 ± 12.4	55.9 ± 15.9	62.3 ± 11.5	0.036
**Clinical features**					
SBP(mmHg)	148.1 ± 44.1	150.3 ± 28.4	159.7 ± 43.6	146.9 ± 32.3	0.124
**Medical history**					
Hypertension	9 (81.8%)	44 (60.3%)	20 (60.6%)	70 (57.4%)	0.739
PCI	1 (9.1%)	14 (19.2%)	2 (6.1%)	25 (20.5%)	0.052
**Laboratory data**					
Neutrophil (10^9^/L)	6.9 (4.9–9.9)	5.3 (4.1–6.8)	5.7 (4.1–9.4)	4.9 (3.6–6.1)	0.079
Lymphocyte (10^9^/L)	1.8 (1.5–2.7)	1.6 (1.3–2.2)	2.0 (1.7–3.1)	1.9 (1.3–2.8)	0.407
Hemoglobin (g/L)	153.2 ± 21.5	143.4 ± 18.1	143.7 ± 18.6	143.5 ± 21.9	0.968
Platelet (10^9^/L)	226 (157–237)	210 (177–251)	190 (156–232)	212 (178–261)	0.040
NT-proBNP (ng/L)	78 (60–100)	141 (48–335)	121 (67–337)	188 (76–705)	0.163
cTn ratio	–	–	0.43 (0.43–0.43)	1.00 (0.43–2.80)	<0.001
Hs-cTn ratio	0.39 (0.21–0.66)	1.6 (0.8–6.4)	–	–	–
Albumin (g/L)	40.9 ± 3.8	40.9 ± 3.6	41.3 ± 4.2	41.9 ± 3.8	0.456
ALT (U/L)	17 (15–29)	21 (14–33)	20 (14–30)	21 (13–31)	0.888
AST (U/L)	19 (18–29)	20 (16–25)	22 (17–27)	21 (17–26)	0.645
BIL (umol/L)	8.9 (7.1–17.5)	8.3 (6.6–10.9)	12.3 (9.3–14.6)	9.0 (6.1–13.1)	0.003
Cr (umol/L)	88 (67–103)	73 (64–85)	83 (72–99)	74 (63–89)	0.033
BUN (mmol/L)	6.9 (6.1–9.2)	6.0 (4.9–7.5)	5.6 (4.8–6.7)	5.7 (4.6–7.7)	0.770
PTA (%)	111 (94–122)	106 (101–116)	96 (90–107)	100 (92–109)	0.488
APTT (seconds)	31.5 (29.4–35.5)	31.1 (28.9–34.9)	28.7 (26.3–30.9)	31.1 (29.0–34.0)	0.004
Fibrinogen (g/L)	2.7 (2.1–3.0)	3.2 (2.7–3.9)	2.6 (2.3–2.9)	3.2 (2.8–3.7)	<0.001
D-dimer (ng/mL)	1143 (291–3216)	85 (47–173)	1276 (409–3038)	128 (79–206)	<0.001

AAS, acute aortic syndrome; ALT, alanine aminotransferase; APTT, activated partial thromboplastin time; AST, aspartate transaminase; BUN, blood urea nitrogen; Cr, creatinine; BIL, bilirubin; HS-cTn, high-sensitivity cardiac troponin; NSTEMI, non-*ST*-elevation myocardial infarction; NT-proBNP, N-terminal pro-brain natriuretic peptide; PCI, percutaneous coronary intervention; PTA, prothrombin time activity; SBP, systolic blood pressure.

Data were presented as numbers (%), mean ± standard deviation, or median and interquartile range as appropriate. Groups were compared using the Student's t-test, Mann–Whitney U test, or Chi-square test as appropriate.

**Table 2:** Univariate and multivariate analysis for differential risk factors for AAS with chest onset <2 h in the cTn cohort.

	**Univariate analysis**		**Multivariate analysis**	
	**OR** **(95%CI)**	***P*** **-value**	**OR** **(95%CI)**	***P*** **-value**
D-dimer	1.003 (1.002–1.005)	<0.001	1.004 (1.002–1.006)	0.001
Age	0.96 (0.93–0.99)	0.012	0.934 (0.892–0.977)	0.003
Fibrinogen	0.19 (0.08–0.42)	<0.001	0.297 (0.098–0.898)	0.032
BIL	1.11 (1.03–1.19)	0.006		0.274
Platelet	0.99 (0.98–0.99)	0.033		0.705
cTn ratio	0.076 (0.013–0.434)	0.004		0.134
Cr	1.003 (0.997–1.010)	0.283		-
APTT	0.99 (0.95–1.05)	0.955		-

AAS, acute aortic syndrome; APTT, activated partial thromboplastin time; BIL, bilirubin; CI, confidence interval; cTnI, cardiac troponin I; OR, odds ratio.

dimer at the optimal cutoff value of 281 ng/mL was sensitive (85.4%) and of high NPV (94.9%) for discriminating AAS with symptom onset at <2 h, which suggested that D-dimer <281 ng/mL could be the exclusion of AAS with onset time at <2 h. Moreover, D-dimer <281 ng/mL with failure rates of 3.0% in AAD (1/33) and 45.5% in IMH (5/11) showed favorable AAD ruled-out properties. Noteworthily, a low sensitivity of 59.1% of D-dimer at the predetermined cutoff level of 500 ng/mL was insufficiently sensitive to exclude AAS, whereas a remarkably high specificity of 96.9% and a PPV of 86.5% were suggestive of a good “rule in” tool for AAS with symptom onset at <2 h (Table 3). A remarkably high specificity (100%) of cTn ratio >1.5 for NSTEMI with symptom onset at <2 h indicated a high probability of NSTEMI, even though the cTn ratio could not be a differential risk factor for discriminating between AAS and NSTEMI with early onset (Table 3).

**Table 3:** Diagnostic performance of D-dimer for AAS and cTn ratio for NSTEMI with chest pain onset <2 h.

	**Threshold**	**Sensitivity (%)**	**Specificity (%)**	**PLR**	**NLR**	**PPV (%)**	**NPV (%)**
D-dimer (ng/mL)	281	86.4	91.3	9.91	0.15	76.8	95.3
	500	59.1	96.9	19.20	0.42	86.5	87.7
cTn ratio	1.0	47.5	93.9	7.84	0.56	–	–
	1.5	41.0	100.0	–	0.59	–	–

AAS, acute aortic syndrome; cTn, cardiac troponin; NLR, negative likelihood ratio; NPV, negative predictive value; NSTEMI, non-*ST*-elevation myocardial infarction; PLR, positive likelihood ratio; PPV, positive predictive value.

### Construction and validation of a nomogram for discriminating AAS from NSTEMI with chest pain onset <2 h

**Figure 2:**
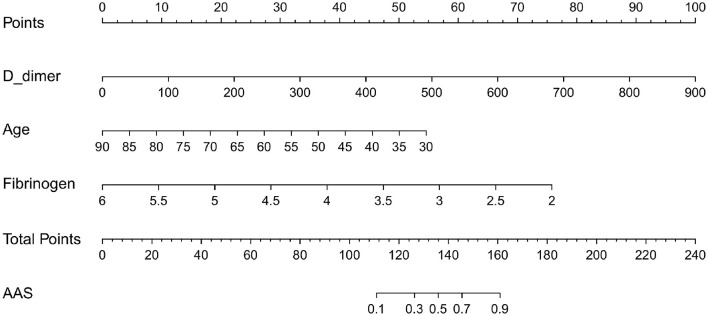
The nomogram model used for discriminating AAS from NSTEMI with chest pain onset at ≥2 h. D-dimer/(ng/mL) >840 calculated as 840; fibrinogen (g/L) <2 calculated as 2; age (years) <32 calculated as 32. AAS, acute aortic syndrome; NSTEMI, non-S*T-*elevation myocardial infarction.

**Figure 3:**
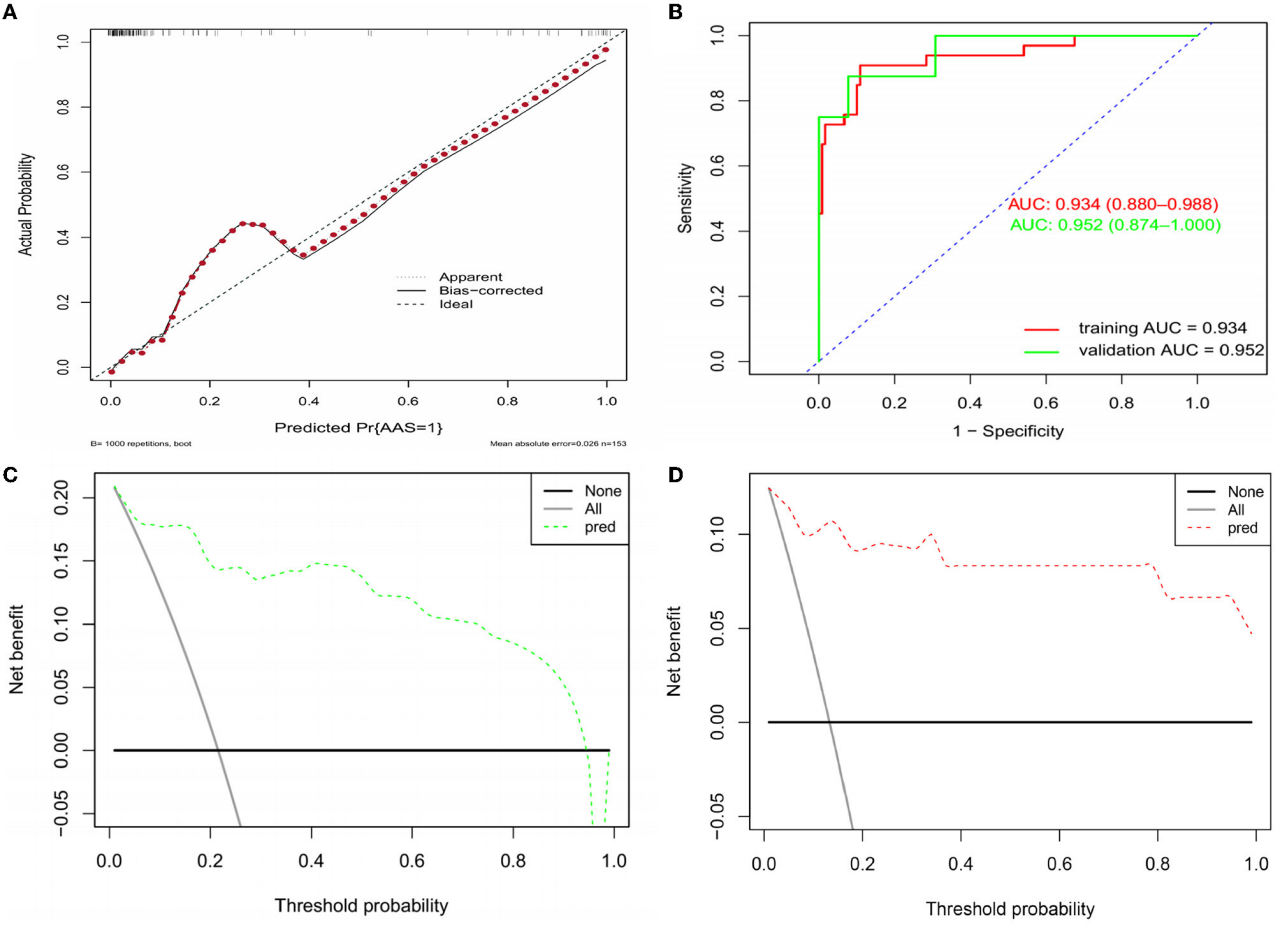
Assessment and validation of the nomogram model. **(A)** Calibration plot in the training set. **(B)** ROC curves in both the training and validation sets. **(C)** Decision curve in the training set. **(D)** Decision curve in the validation set. AUC, areas under the curve; ROC, receiver operating characteristic.

Based on the independent predictors (D-dimer, fibrinogen, and age) for AAS with symptom onset at <2 h in the cTn cohort, a full nomogram was established to predict the risk of AAS (Figure 2). Regression coefficients of the variables were correspondingly converted to scores within a range of 0–100, reflecting their relative importance. In the diagnosis of AAS, D-dimer/(ng/mL) >836, fibrinogen/(g/L) <2, age/(years) <32 all have specificities of >99%, and hence the upper limit of D-dimer/(ng/mL) was set as 836 and the lower limits of fibrinogen (g/L) and age (years) were set as 2 and 32, respectively, in the construction of the nomogram. The probability of AAS in a patient can be calculated by summing the points of all these factors. The full nomogram was validated in the HS-cTn cohort. The calibration plot displayed an overall good agreement between the prediction of the nomogram model and the actual probability of AAS (Figure 3A). The ROC curves were drawn to evaluate the predictive performance in both sets (Figure 3B). The AUC was 0.934 (95% CI, 0.880–0.988) in the training set and 0.952 (95% CI, 0.874–1.000) in the validation set, respectively. To assess the clinical utility, decision curve analyses were performed in both sets (Figures 3C, D).

### Demographic characteristics and analysis for AAS patients and NSTEMI patients with chest pain onset ≥2 h

A total of 95 patients with AAS (80 patients with AAD, 14 patients with IMH, and 1 patient with PAU) and 167 patients with NSTEMI were included in the cTn cohort. Accordingly, 39 patients with AAS (36 patients with AAD, 3 patients with IMH) and 98 patients with NSTEMI were included in the HS-cTn cohort (Table 4). Univariate analysis identified that D-dimer, neutrophil, BIL, platelet, creatinine, hemoglobin, age, PTA, fibrinogen, lymphocyte, and percutaneous coronary intervention incidence rate were significantly different between AAS and NSTEMI in the cTn cohort (*P* < 0.05). Multivariate analysis revealed that D-dimer, neutrophil, BIL, and platelet were the differential risk factors for AAS compared with NSTEMI (*P* < 0.05) (Table 5).

**Table 4:** Baseline characteristics of AAS vs NSTEMI with chest pain onset ≥2h.

**Characteristic**	**HS-cTnI cohort** (***N** =* **137)**		**cTnI cohort** (***N** =* **262)**		
	**AAS (*****N** =* **39)**	**NSTEMI(*****N** =* **98)**	**AAS (*****N** =* **95)**	**NSTEMI(*****N** =* **167)**	***P*** **-value**
**Demographic data**					
Male sex	32 (82.1%)	63 (64.3%)	70 (73.7%)	104 (62.3%)	0.143
Age (years)	57.1 ± 11.7	62.4 ± 13.0	55.4 ± 12.6	63.6 ± 12.0	<0.001
**Clinical features**					
SBP (mmHg)	161.0 ± 36.2	156.6 ± 28.8	155.3 ± 38.6	153.7 ± 31.2	0.720
**Medical history**					
Hypertension	29 (74.4%)	68 (69.4%)	68 (71.6%)	105 (62.9%)	0.153
PCI	1 (2.6%)	26 (26.5%)	2 (2.1%)	25 (15.0%)	0.001
**Laboratory data**					
Neutrophil (10^9^/L)	9.6 (7.8–12.9)	5.8 (4.7–7.7)	10.7 (8.1–13.1)	5.7 (4.4–7.9)	<0.001
Lymphocyte (10^9^/L)	0.9 (0.6–1.1)	1.4 (1.1–2.0)	1.1 (0.7–1.5)	1.6 (1.1–2.1)	<0.001
Hemoglobin (g/L)	141.1 ± 16.2	141.0 ± 20.1	136.5 ± 18.1	141.4 ± 19.5	0.046
Platelet (10^9^/L)	172 (140–212)	207 (176–259)	184 (150–220)	213 (174–244)	<0.001
NT-proBNP (ng/L)	315 (114–886)	188 (71–608)	238 (127–658)	196 (86–655)	0.198
cTn ratio	–	–	0.43 (0.43–0.56)	2.18 (0.83–6.96)	<0.001
Hs-cTn ratio	0.71 (0.34–2.39)	4.6 (2.2–10.6)	–	–	–
Albumin (g/L)	41.0 ± 4.3	41.7 ± 3.6	41.4 ± 4.7	41.7 ± 4.1	0.674
ALT (U/L)	17 (14–24)	18 (13–29)	19 (13–31)	18 (13–28)	0.847
AST(U/L)	20 (16–24)	20 (16–30)	21 (16–27)	22 (17–28)	0.531
BIL (umol/L)	16.0 (11.2–21.5)	8.3 (6.5–12.3)	13.9 (9.1–18.2)	9.2 (6.9–11.3)	<0.001
Cr (umol/L)	81 (64–108)	68 (59–79)	79 (61–99)	69 (58–82)	0.005
BUN (mmol/L)	6.3 (5.1–7.7)	6.1 (4.9–7.6)	6.3 (5.0–8.1)	6.2 (4.9–7.4)	0.275
PTA (%)	94 (85–101)	105 (97–113)	91 (84–98)	101 (93–108)	<0.001
APTT (seconds)	32.0 (30.0–34.3)	32.0 (29.3–34.0)	30.0 (28.0–32.0)	30.0 (27.9–32.2)	0.707
Fibrinogen (g/L)	2.8 (2.1–3.3)	3.1 (2.8–3.7)	2.7 (2.2–3.3)	3.1 (2.8–3.6)	<0.001
D-dimer (ng/mL)	1,371 (610–6,324)	110 (48–192)	1,265 (661–3,243)	126 (76–210)	<0.001

AAS, acute aortic syndrome; ALT, alanine aminotransferase; APTT, activated partial thromboplastin time; AST, aspartate transaminase; BUN, blood urea nitrogen; Cr, creatinine; BIL, bilirubin; HS-cTn, high-sensitivity cardiac troponin; NSTEMI, non-*ST*-elevation myocardial infarction; NT-proBNP, N-terminal pro-brain natriuretic peptide; PCI, percutaneous coronary intervention; PTA, prothrombin time activity; SBP, systolic blood pressure.

Data were presented as numbers (%), mean ± standard deviation, or median and interquartile range as appropriate. Groups were compared using the Student's t-test, Mann–Whitney U test, or Chi-square test as appropriate.

**Table 5:** Univariate and multivariate analysis for differential risk factors for AAS with chest pain onset ≥2h in the cTn cohort.

	**Univariate analysis**		**Multivariate analysis**	
	**OR** **(95%CI)**	***P*** **-value**	**OR** **(95%CI)**	***P*** **-value**
D-dimer	1.003 (1.002–1.004)	<0.001	1.003 (1.002–1.004)	<0.001
Neutrophil	1.60 (1.42–1.80)	<0.001	1.53 (1.28–1.83)	<0.001
BIL	1.15 (1.09–1.21)	<0.001	1.20 (1.08–1.33)	0.001
Platelet	0.99 (0.99–1.00)	<0.001	0.99 (0.98–1.00)	0.018
Cr	1.01 (1.00–1.02)	0.018		0.066
Hemoglobin	0.99 (0.97–1.00)	0.048		0.729
Age	0.95 (0.93–0.97)	<0.001		0.070
PTA	0.91 (0.90–0.94)	<0.001		0.678
Fibrinogen	0.57 (0.40–0.80)	0.001		0.902
Lymphocyte	0.35 (0.22–0.56)	<0.001		0.912
PCI	0.12 (0.03–0.53)	0.005		0.094
cTn ratio	1.003 (0.997–1.008)	0.376		–

AAS, acute aortic syndrome; CI, confidence interval; Cr, creatinine; cTn, cardiac troponin; BIL, bilirubin; OR, odds ratio; PTA, prothrombin time activity; SBP, systolic blood pressure.

D-dimer at a cutoff level of 385 ng/mL was the threshold leading to the maximum summation of sensitivity of 91.8% and specificity of 91.3% in discriminating AAS from NSTEMI with symptom onset at ≥2 h. Compared with a relatively low sensitivity of D-dimer at a level of 500 ng/mL, D-dimer <385 ng/mL was more appropriate as negativity due to the reduction in the misdiagnosis of AAS as NSTEMI (Table 6). Both high specificity (93.7%) of cTn ratio >5 and high specificity (97.4%) of HS-cTn ratio >15 for NSTEMI with symptom onset at **≥**2 h indicated a high probability of NSTEMI (Table 6).

**Table 6:** Diagnostic performance of D-dimer for AAS and cTn ratio for NSTEMI with chest pain onset ≥2 h.

	**Threshold**	**Sensitivity (%)**	**Specificity (%)**	**PLR**	**NLR**	**PPV (%)**	**NPV (%)**
D-dimer (ng/mL)	385	91.8	91.3	10.58	0.09	77.9	97.1
	500	86.6	94.3	15.29	0.14	83.6	95.5
cTn ratio	5	29.3	93.7	4.65	0.75	–	–
	10	18.0	95.0	3.41	0.87	–	–
HS-cTn ratio	10	27.6	89.7	2.69	0.81	–	–
	15	15.3	97.4	6.00	0.85	–	–

AAS, acute aortic syndrome; HS-cTn, high-sensitivity cardiac troponin; NLR, negative likelihood ratio; NPV, negative predictive value; NSTEMI, non-*ST*-elevation myocardial infarction; PLR, positive likelihood ratio; PPV, positive predictive value.

### Construction and validation of a nomogram for discriminating AAS from NSTEMI with chest pain onset ≥2h

**Figure 4:**
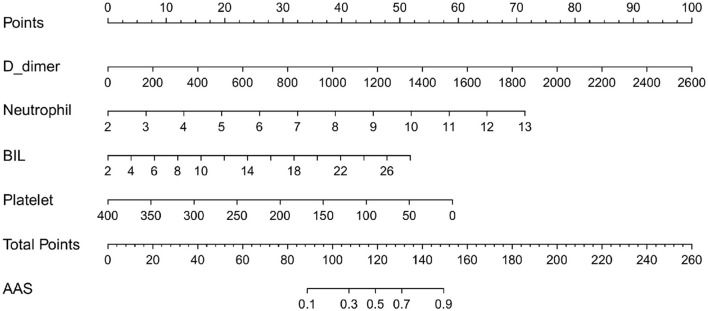
The nomogram model used for discriminating AAS from NSTEMI with chest pain onset at ≥2 h. D-dimer/(ng/mL) >2,520 calculated as 2,520; neutrophil/(10^∧^9/L) >13 calculated as 13; BIL/(U/L) > 27.6 calculated as 27.6. AAS, acute aortic syndrome; BIL, bilirubin; NSTEMI, non-S*T-*elevation myocardial infarction.

**Figure 5:**
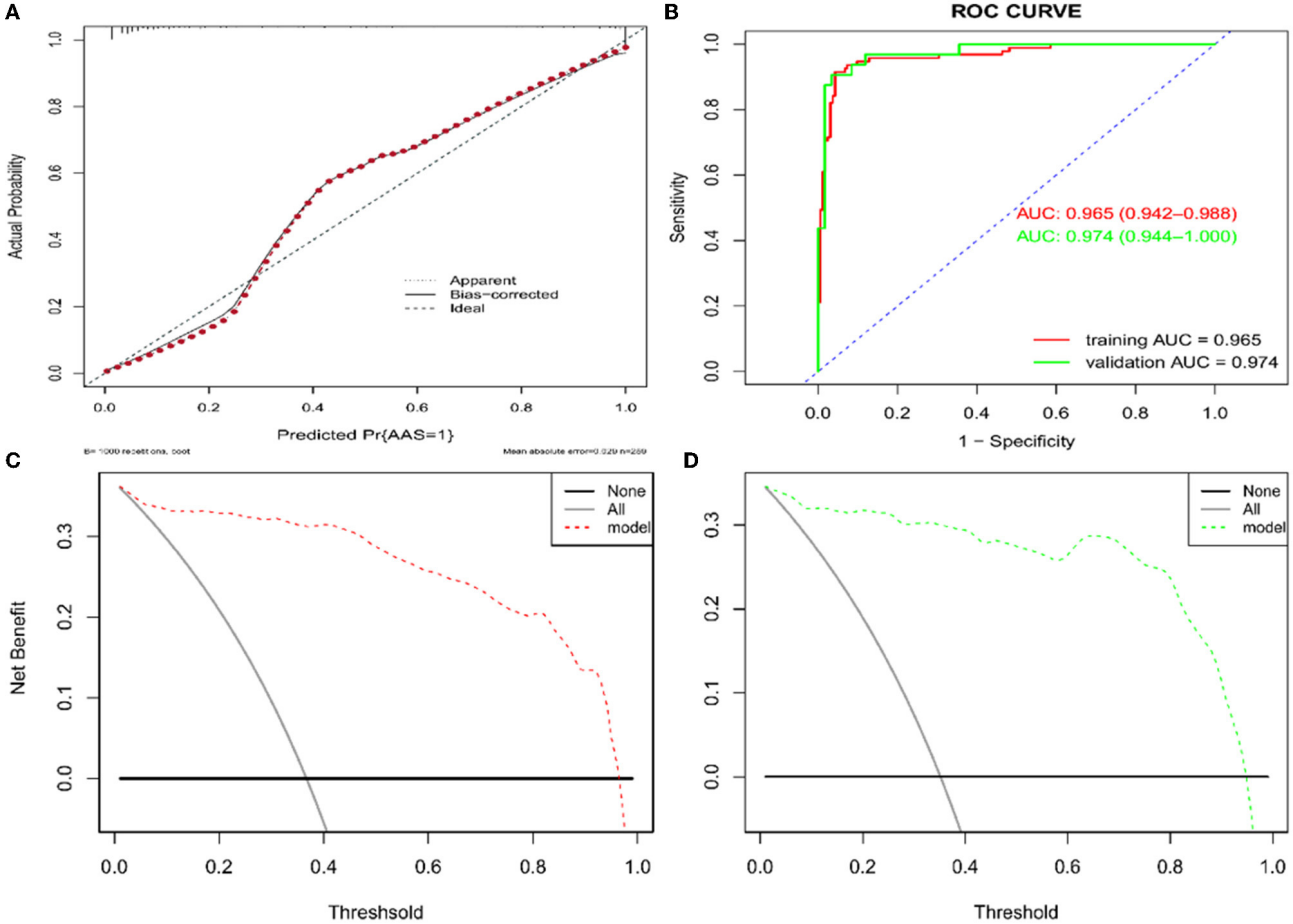
Assessment and validation of the nomogram model. **(A)** Calibration plot in the training set. **(B)** ROC curves in both the training and validation sets. **(C)** Decision curve in the training set. **(D)** Decision curve in the validation set. AUC, areas under the curve; ROC, receiver operating characteristic.

Based on the independent predictors (D-dimer, neutrophil, BIL, and platelet) for AAS chest pain onset at ≥2 h in the cTn cohort, a full nomogram was established to predict the risk of AAS (Figure 4). In the diagnosis of AAS, D-dimer/(ng/mL) >2,520, neutrophil/(10^∧^9/L) >13, BIL/(U/L) >27.6 all have specificities of >99%, and hence the upper limits of D-dimer/(ng/mL), neutrophil/(10^∧^9/L), and BIL/(U/L) were set as 2,520, 13, 27.6, respectively, in the construction of the nomogram. The AUC was 0.965 (95% CI, 0.942–0.988) in the training set and 0.974 (95% CI, 0.944–1.000) in the validation set, respectively (Figure 5B). No significant difference was found between the two AUCs (*P* = 0.623). Based on net benefit results of the decision curves in both sets and clinical practice, we chose the probabilities of AAS as 0.5 and 0.9 calculated by the nomogram model as the cutoff values, and the estimated probabilities of <0.5 were considered as low-risk of AAS, 0.5–0.9 as intermediate-risk of AAS, and >0.9 as high-risk of AAS, respectively. The low-risk, intermediate-risk, and high-risk groups accounted for 67, 9, and 24% of the patients in the entire cohort, respectively (Figures 5C, D).

### Levels of differential risk factors for discriminating between AAS and NSTEMI at various time points

**Figure 6:**
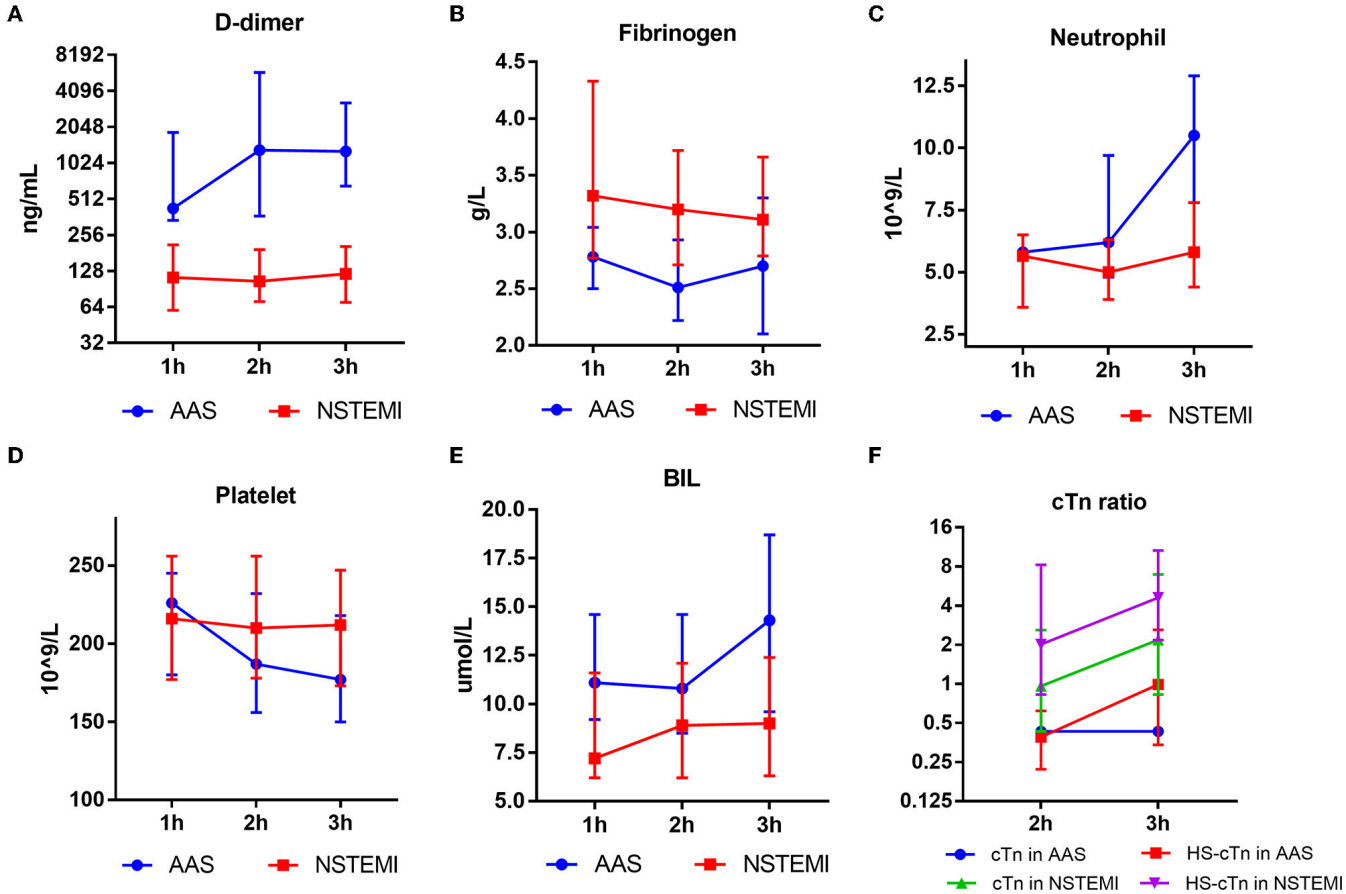
Results of D-dimer **(A)**, fibrinogen **(B)**, neutrophil **(C)**, platelet **(D)**, BIL **(E)**, cTn ratio **(F)** of AAS and NSTEMI at different time points. AAS, acute aortic syndrome; BIL, bilirubin; HS-cTn, high-sensitivity cardiac troponin; NSTEMI, non-S*T-*elevation myocardial infarction.

D-dimer of AAS displayed an increase from 1 h of onset, and the platelet of AAS showed an opposite trend from 2 h of onset. In contrast, the D-dimer and platelet of NSTEMI remained almost at the same level. The fibrinogen of AAS decreased and reached the lowest point at 2 h of onset; however, the fibrinogen of NSTEMI displayed a gradual but minor extent of decrease compared with that of AAS. Significant discrepancies of neutrophil and BIL between AAS and NSTEMI were observed from 2 h of onset (Figure 6).

## Discussion

Our study found that D-dimer, fibrinogen, and age were the differential risk factors for discriminating AAS from NSTEMI with chest pain onset at <2 h, and as for patients with symptom onset at ≥2 h, D-dimer, neutrophil, BIL, and platelet were the differential risk factors for AAS. Both nomogram models were established and validated to predict the risk of AAS under specific conditions.

D-dimer, a degradation fragment of plasma fibrin after fibrinolysis of thrombus (7), is well established as a diagnostic biomarker for AAD (5, 8). Gorla et al. demonstrated that a low score in aortic dissection detection risk score (ADD-RS) combined with D-dimer of <500 ng/mL could effectively exclude the diagnosis of AAS (9). However, Salmasi et al. found that only 26.8% of ED in clinical terms would consider applying the AAS diagnosis flowchart to patients with chest pain (10). Furthermore, a negative level of D-dimer of <500 ng/mL was not sufficient to exclude all AAS (11–14) and the negative rate of D-dimer of 0.3–4.4% (15). Circulating levels of D-dimer rise within 6 h of AAD onset (16). The diagnostic performance of D-dimer in most previous studies was assessed at the platform levels of D-dimer after 6 h of AAD onset (5). As for a few studies about AAS with early-onset, the positive rate of D-dimer was relatively low within 2 h compared with that beyond 2 h (11), and our results further indicated that the shorter the time course from symptom onset, the lower optimal cutoff level of D-dimer should be set. Meta-analysis suggested that the negative D-dimer result useful to rule out AAD was only limited in patients with low risk in ADD-RS (13), and patients with IMH tend to have lower D-dimer levels than patients with AAD due to less extension of the disease (17), and thus, besides early-onset time, the inclusion of patients with moderate and high risk in ADD-RS and patients with IMH in our study also contributed to the low cutoff level of D-dimer. The specificity of D-dimer for diagnosis of AAS varies greatly among different studies. A systematic review reported that D-dimer at a level of 500 ng/mL for AAS ranged from 32.8 to 89.2% specific among 6 studies (18). The high specificity of D-dimer of 500 ng/mL in our study, especially for the diagnosis of AAS with onset time <2 h, can largely be explained by the absence of other diseases with high D-dimer levels, such as pulmonary embolism (PE).

Acute aortic syndrome is a disease of inflammation and coagulation disorders (19–21). Higher D-dimer and white cell count were found in AAS than those in NSTEMI, a result consistent with previous findings (8), and an increment in neutrophils was observed in our results as early as 1 h after onset. The decreased platelet count and fibrinogen of AAS in our study could be interpreted by the excessive consumption in response to thrombosis of the false lumen (20, 21). In addition to hemostatic function, fibrinogen is an acute phase reactant (21), which probably explained why the fibrinogen of AAS in our study was higher beyond 2 h than that within 2 h. Approximately 4.8–34% of AAD are complicated with malperfusion (22). Elevated BIL of AAS could be attributed to liver dysfunction (secondary to liver malperfusion or inflammatory damage) and/or hemolysis. Due to the hepatic function of AAS comparable with those of NSTEMI, we speculated that elevated BIL of AAS was more ascribed to hemolysis. The discrepancy of differential risk factors for various onset times not only indicated a higher extent of activation of inflammatory and thrombosis system in the large area of aorta injury of AAS than in the small or medium vessels of NSTEMI but also the time-dependent quality of the different pathological process.

Elevated troponin was observed in 25% of patients with AAD (23) and up to 54% if HS-cTn was employed, which led to in-hospital diagnostic delay (24). Similarly, elevated cTn was observed in 19.6% of AAS, and up to 36% if using the high-sensitivity assay in our results. The mechanisms of elevated troponin in AAS include coronary ostia dissection or occlusion by the flap, acute left ventricular compromise leading to aortic insufficiency, and chronic microvascular disease (24). Previous studies observed that troponin positivity could not be the predictor of misdiagnosis of AAS (25, 26), and our study found that elevation of cTn ratio could not be a differential risk factor for AAS and NSTEMI with early onset. However, both high specificities of cTn ratio >1.5 for NSTEMI with onset at <2 h and cTn ratio >5 for NSTEMI with onset at **≥**2 h implied that the diagnostic performance of cTn relied on the extent to which cTn was elevated and the onset time. Of note, a relatively low specificity of HS-cTn ratio >10 for NSTEMI indicated that clinicians should be cautious to exclude the diagnosis of AAS, as proposed by guidelines to implement 0/1 or 0/2 h algorithm to observe the absolute changes of HS-cTn (4), instead of judging by one blood test result, which may avoid misdiagnosis of AAS as NSTEMI.

The strength of our study was the development and validation of nomogram models for quantitatively evaluating the risk of AAS compared with NSTEMI. Although biomarkers such as smooth muscle myosin heavy chain (27), calponin (27), or soluble ST2 (6) exhibited diagnostic performance for AAS, the inaccessibility of these biomarkers limited routine usages. The nomogram models comprised standard laboratory biomarkers that could be regarded as practical and reliable approaches applied to patients with initial suspicion of AAS and provided risk evaluation for patients with AAS with relatively low or negative D-dimer results.

## Limitations

Our study had certain limitations that should be emphasized.

First, PE, myocarditis, pericarditis, sepsis, pneumothorax, and other diseases presenting with chest pain were not included in this study, and thus, our study could not be an adequate representation of the clinical scenario of chest pain in the emergency room, and our result could not be interpreted as the differentiation of AASs from any patient with chest pain.

Second, confined by the nature of the retrospective analysis, a healthy group as the control group was not reported in our study, therefore, the results of sensitivity and specificity of D-dimer in a selected population of NSTEMI and AASs may not be valid in the clinical scenario of chest pain in the emergency room.

Third, all patients enrolled in our study belonged to the Asian population, and ethnicity *per se* may affect the level of D-dimer (28); thus, our results may be not represent other ethnic groups.

Fourth, echocardiography represents an essential diagnostic tool for AASs (3) and some other diseases such as pericarditis and has the advantage of being widely available, cos*t-*effective, noninvasive, and well-tolerated. However, echocardiographic data were not included in the diagnostic model and might influence the efficacy of the diagnostic model to some extent. A diagnostic model integrating clinical data, biomarkers, and imaging tools to predict the differential diagnosis of chest pain will be the direction of future research.

Finally, this was a retrospective single-center study with a limited number of patients. Therefore, a large-scale multicenter prospective clinical trial adequately representing the clinical scenario of chest pain in the emergency room is warranted to further distinguish these diseases during the troponin-blind period.

## Conclusion

The time-dependent quality of D-dimer should be considered in terms of discriminating AAS from NSTEMI. A shorter time course from symptom onset and a lower cutoff level of D-dimer should be set to rule out AAS. The nomogram models have clinical utility in the risk evaluation of AAS compared with NSTEMI with early onset time.

## Data availability statement

The raw data supporting the conclusions of this article will be made available by the authors, without undue reservation.

## Ethics statement

The studies involving human participants were reviewed and approved by Shengjing Hospital of China Medical University Ethics Review Board. The patients/participants provided their written informed consent to participate in this study.

## Author contributions

ZS designed the study and supervised the enrollment from which patient data in this work was generated. YW performed all statistical analysis, developed, and validated the nomogram models. ZS and FT wrote the manuscript. All authors read and approved the final manuscript.
